# Characterization and Simulation of the Interface between a Continuous and Discontinuous Carbon Fiber Reinforced Thermoplastic by Using the Climbing Drum Peel Test Considering Humidity

**DOI:** 10.3390/polym16070976

**Published:** 2024-04-03

**Authors:** Nicolas Christ, Benedikt M. Scheuring, Christoph Schelleis, Wilfried V. Liebig, John Montesano, Kay A. Weidenmann, Jörg Hohe

**Affiliations:** 1 Institute for Applied Materials, Karlsruhe Institute of Technology, 76131 Karlsruhe, Germany; benedikt.scheuring@kit.edu (B.M.S.); wilfried.liebig@kit.edu (W.V.L.); 2 Component Safety and Lightweight Construction, Fraunhofer Institute for Mechanics of Materials, 79108 Freiburg, Germany; joerg.hohe@iwm.fraunhofer.de; 3 Polymer Engineering, Fraunhofer Institute for Chemical Technology ICT, 76327 Pfinztal, Germany; christoph.schelleis@ict.fraunhofer.de; 4 Lightweight Design, Institute of Vehicle Systems Technology, Karlsruhe Institute of Technology, 76131 Karlsruhe, Germany; 5 Department of Mechanical and Mechatronics Engineering, Univesity of Waterloo, Waterloo, ON N2L 3W8, Canada; john.montesano@uwaterloo.ca; 6 Institute of Materials Resource Management, University of Augsburg, 86159 Augsburg, Germany; kay.weidenmann@mrm.uni-augsburg.de

**Keywords:** carbon fibers, polyamide 6, layered structures, delamination, cohesive interface modeling, mechanical testing, continuous-discontinuous FRP

## Abstract

The objective of this paper is to investigate the debonding behavior of the interface between continuously and discontinuously fiber reinforced thermoplastics using the climbing drum peel test. The study emphasizes on the importance of considering different climatic boundary conditions on the properties of thermoplastics. Specimens with varying moisture contents, from 0m.% up to above 6m.% are prepared and tested. It is observed that an increase in moisture content from 0m.% to 2m.% results in an increase of the fracture surface energy from $1.07\cdot 10^{3}\;J/m^{2}$ to $2.40\cdot 10^{3}\;J/m^{2}$ required to separate the two materials, but a further increase in moisture to 6.35m.% conversely results in a subsequent decrease of the required energy to $1.91\cdot 10^{3}\;J/m^{2}$. The study presents an explanatory model of increasing plasticization of the polymer due to increased polymer chain mobility, which results in more deformation energy being required to propagate the crack, which is corroborated in SEM investigations of the fracture surface. A further increase in humidity leads to polymer degradation due to hydrolysis, which explains the subsequent reduction of the fracture energy. The experimental set up is modeled numerically for the first time with cohesive surfaces, which could successfully reproduce the effective force-displacement curve in the experiment by varying the interface parameters in the model over an influence length, allowing the conclusion of a process induced variation in the interface properties over a specific consolidation length.

## 1. Introduction

Fiber reinforced thermoplastic polymers (FRTPs) have gained significant attention in recent years, owing to their remarkable mechanical properties, lightweight nature, and ease of processing [1]. These composites offer enhanced density specific strength, stiffness, and impact resistance, rendering them highly desirable for applications across various industries such as automotive, aerospace, and sporting goods [2]. In light of global challenges regarding overall resource consumption [3] the inherent recyclability of thermoplastic polymers represents a decisive advantage over thermoset polymers [4]. Performance of FRTPs hinges on interface quality and integrity between reinforcing fiber and matrix [5,6]. Adding different types of fiber reinforcements, e.g., continuous (Co) and discontinuous (Dico) reinforcement, the CoDico hybrid composite also depends on the interface quality between constituents [7,8].

This research study is centered on compression molding, characterization and simulation of carbon fiber (CF) reinforced polyamide 6 (PA 6) CoDico interfaces between long fiber thermoplastic (LFT) molding compounds and unidirectional (UD) tapes. Carbon fibers, renowned for their exceptional mechanical properties, high stiffness, and low weight, are an ideal choice for reinforcing thermoplastics [9]. Using Co CF provides superior strength and stiffness to the composite, while Dico CF offer cost advantages, high design freedom and improved impact resistance without compromising a significant portion of the strength properties [10,11]. LFT molding compounds can be processed in the LFT direct (LFT-D) inline process [12]. Here, a wide variety of matrix polymers and reinforcing fibers can be compounded and compression molded in one efficient processing step from raw materials to finished part [13]. Compression molded LFT-D composites are destined for high volume production and are a staple in industry production [9,14]. A process overview and description of the LFT-D material portfolio has recently been given by Schelleis et al. [15]. Work with PA 6 CF and PA 66 CF specifically was presented by Bondy et al. and Dahl et al. [16,17], respectively. Irregardless of design, interface quality between Co and Dico FRTP determines overall performance and reliability of CoDico materials. Local tailored LFT-D was developed to reinforce global PA 6 CF Co materials by local application of PA 6 GF LFT-D Dico materials in the research project SMiLE. Interface characterizations were done to optimize co-molding regarding heating temperatures of UD tape especially. It was found that tape temperatures lower than 80 °C result in low interfacial shear strength, while increasing temperatures to 130 °C and 275 °C lead to an increase in interfacial shear strength [18,19,20]. Kugele formulated a lower process boundary for tape surface temperatures while overmolding at the crystallization temperature of PA 6 at around 180 °C. This boundary can be crossed using thick laminates that store the energy during transfer or if the overmolding phase transfers sufficient energy into the interface during overmolding [21]. Interface quality significantly influences crucial properties such as interfacial adhesion, load transfer efficiency, and resistance to environmental factors [22,23]. Understanding and characterizing the behavior of the CoDico interface under various conditions is essential for optimizing the design and performance of FRTPs.

Among the challenges associated with interface characterization, the influence of environmental factors, particularly humidity, is of immense importance. Moisture absorption in FRTPs can trigger various degradation mechanisms, including fiber-matrix debonding, polymer swelling, matrix plasticization, and altered interfacial adhesion, which ultimately impact the composite’s mechanical properties [24,25,26]. Therefore, it becomes imperative to evaluate the behavior of the interface considering the effect of humidity to ensure the composite’s performance under real-world conditions. Being a hygroscopic polymer, polyamide 6 (PA 6) tends to absorb water [27,28]. The diffusion and absorption of water in PA 6 is thereby governed by specific molecular interactions between the polymer and water molecules. In particular, water tends to penetrate primarily into the amorphous regions between the crystalline domains, where it plasticizes the polymer and increases chain mobility. The rate of moisture transport is significantly influenced by both the ambient moisture concentration and the ambient temperature. Fick’s diffusion equation is often used to model water diffusion in PA 6 due to its simplicity and effectiveness [29]. This model characterizes the change in mass over time by a diffusion coefficient, for which the temperature dependence is commonly described by the Arrhenius equation [27].

The climbing drum peel (CDP) test is employed as a valuable method for evaluating the interfacial strength and durability of FRTPs [30]. This test provides insights into the energy required to separate the CoDico interface and facilitates the assessment of interfacial adhesion properties. One of the earliest study to investigate the CDP test on glass fiber reinforced laminates can be found in [31]. By conducting the climbing drum peel test under varying humidity conditions, a comprehensive understanding of the interface behavior and its influence on the overall composite performance can be achieved. Historically, the climbing drum peel test has been predominantly used to assess the interfacial adhesion between different materials in sandwich structures, such as metals, plastics, and adhesives. However, its application to the characterization of the interface between CoDico FRTPs is a novel approach. Other research on FRP using the CDP test can be found in [32], where in the interphase bond strength between Co bonded thermoplastic-thermoset hybrid composites was investigated. Furthermore, in [33] the interlaminar mode I fracture toughness within thin Co laminates was investigated.

The advantage of the CDP test over the better known double cantilever beam (DCB) test is that the crack front correlates directly with the position of the drum, which means that the crack front does not have to be determined separately [34]. Furthermore, and most importantly, the CDP test allows the characterization of asymmetric structures and specimens, which is usually the case for CoDico hybrids due to the inherent thin layer of Co reinforcement. Our first approach to investigate the interface was the roll shear test according to DIN ISO 1464, in which the Co layer is deflected around a 25 mm diameter roll and thus sheared by the Dico. However, preliminary tests showed that the radius was too small for unidirectional CF tapes and that the high bending stress caused the Co layer to fracture before it could be sheared off.

The subject of this work is to characterize the interfacial properties, assess the influence of humidity on the adhesion strength, and simulate the behavior of the interface using computational models. The outcomes of this study will contribute to the optimization of design parameters for fiber-reinforced thermoplastics, facilitating the development of robust and reliable composite materials for diverse applications.

## 2. Climbing Drum Peel Test

**Figure 1:**
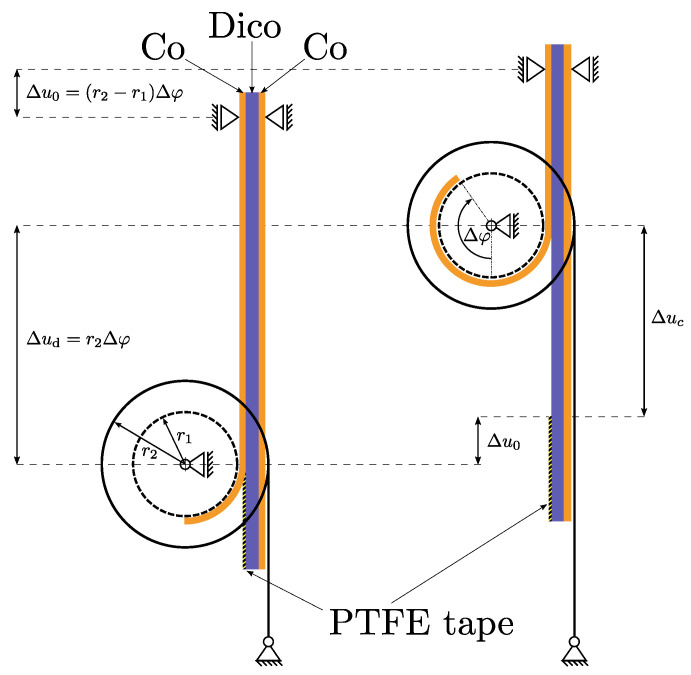
Kinematics of the CDP test in reference to [34]. $\Delta u_{0}$, $\Delta u_{d}$ and $\Delta u_{c}$ are the increments of the prescribed displacement at the upper clamp, the displacement of the drum with respect to its initial position and the crack length with respect to the structure, respectively. Δφ is the rotation angle increment of the drum and $r_{1}$ and $r_{2}$ are the inner and outer radii of the drum, respectively.

According to DIN EN 2243-3 [35] and ASTM D1781 [36], the climbing drum peel test was developed to characterize the adhesive bond between two materials. In general, the adhesive interface under investigation is between a relatively flexible and a rigid structure, where the flexible structure or peel arm should be wrapped around the climbing drum. Thereby, the interface is tested in mode I. An illustration of the test is given in Figure 1. Overall, the CDP method has a distinct advantage over more conventional techniques such as the DCB test, a fact that has been well documented in the existing literature. Owing to testing kinematics, a separate crack detection as in the well-known DCB experiment is not necessary, since the crack position directly correlates with the current drum position. An added benefit is that, unlike the DCB test, the CDP can evaluate asymmetric structures without causing the crack to deviate from the intended plane [33]. Furthermore, Daghia et al. were able to corroborate another anticipated advantage of the CDP test over the DCB through X-ray computed tomography, specifically regarding the distinction between the straight and curved shapes of the crack front [34]. In addition, the variability observed in the peel test data is significantly less than the variability observed in Mode I fracture toughness tests on DCB specimens [33]. In its common form, the experiment is evaluated to extract the average peel torque. Daghia et al. expanded the evaluation to further extract information about the critical strain energy release rate [34]. For this, the authors state three requirements for the experimental setup:

- the radius of the drum needs to be large enough,
- the peel arm needs to be flexible,
- the winding forces need to be large,

without further specifying numeric values. The reason for these requirements is that a structure that does not wrap coherently around the drum, e.g., a structure with a low flexural modulus, violates the energetic assumptions in the analysis and thus prevents the evaluation of material parameters. A detailed explanation on this matter is given in the appendix in [34].

For the test, the upper part of a specifically prepared specimen is clamped to the test machine. The lower side of the specimen includes a pre-crack (details on the specimen preparation are given in Section 3), causing a free end of Co layer to be peeled from the rest of the specimen. The flexible, free end of the Co layer is attached to the drum at the smaller radius $r_{1}$. Two loading straps are wrapped around the second radius of the drum $r_{2}$ and are connected to ground, which apply the necessary torque on the drum to delaminate the interface and propagate the crack. The torque is induced once the test starts when a constant displacement rate is prescribed to the clamping on top. Owing to the kinematics of the test, the displacement ratio of the drum center $\Delta u_{d}$ to the clamping $\Delta u_{0}$ is given by

(1) $$\frac{\Delta u_{d}}{\Delta u_{0}}=\frac{r_{2}}{r_{2}-r_{1}},$$

and since $r_{2}>r_{1}$ this ratio is always larger than one making the drum ’climb’ upwards. The relative crack propagation $\Delta u_{c}$, or newly formed crack length, can be calculated at every point in time with

(2) $$\Delta u_{c}=\Delta u_{d}-\Delta u_{0}=\frac{r_{1}}{r_{2}-r_{1}}\Delta u_{0}.$$

**Figure 2:**
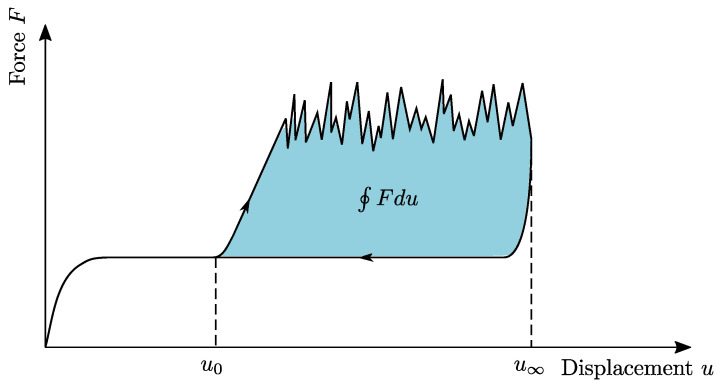
Schematic force-displacement diagram of a CDP test for testing the interface of CoDico FRPs.

A schematic force-displacement diagram including the loading and unloading phase of the CDP test is given in Figure 2. The work done to separate the interface can easily be calculated from the force-displacement diagram

**Figure 3:**
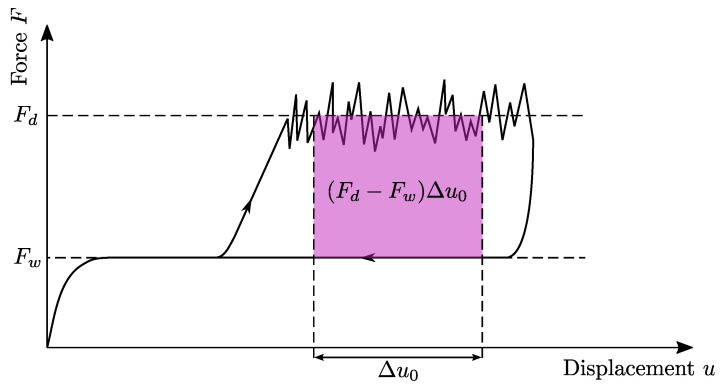
Schematic force-displacement diagram of a CDP test for testing the interface of CoDico FRPs with an average work approach.

(3) W=∮Fdu,

where also the initial effects in the rise of the force signal are considered. To avoid initial and ending effects of the force signal, one can also use an average approach, indicated in Figure 3, and using the following relation  

(4) $$W=\left(F_{d}-F_{w}\right)\Delta u_{0},$$

where $F_{d}$ is the average delamination force and $F_{w}$ is the average winding force, which is caused by the weight of the drum. The measure of interest, i.e., the critical strain energy release rate $G_{c}$, can then be calculated by dividing the work done to separate the interface by the area created by the propagation of the crack. For Equation (3), i.e., when the whole separation process is considered, the created surface can either be measured from the separated specimen or calculated by using $\Delta u_{c}$ from Equation (2) multiplied by the width of the specimen *w*. When only a fraction of the created surface is used, as is the case in Equation (4), a measurement is not feasible and the created surface needs to be calculated. The critical energy release rate is then calculated with

(5) $$G_{c}=\frac{W}{w\Delta u_{c}},$$

which is in agreement with [37]. Care has to be taken that $\Delta u_{c}$ can either be the full crack length, for when the full work (cf. Equation (3)) is used, or it can be a partial crack length.

## 3. Materials and Methods

TechnylStar XS 1352 BL PA 6 and matching masterbatch, KNF/2, was provided by DOMO Chemicals GmbH (Leuna, Germany) and used as LFT-D matrix material. ZOLTEK PX 35 Tow with sizing for PA 6 was procured from Zoltek Corporation (Bridgeton, MO, USA). For the Co phase TECHNYL LITE C130 C60, a PA 6 CF tape also from DOMO, was used. To make the interface testable, the LFT-D overmolding process was adapted to produce plates with initial cracks (see Section 3.1). Asymmetrically reinforced plates warp, so a double-sided, sandwich-style, reinforcement, was used here.

### 3.1. Production of Semi-Finished Materials and Plates

**Figure 4:**
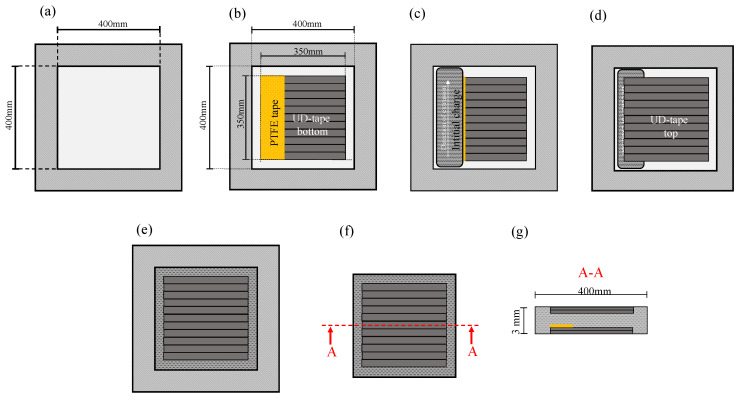
Schematic representation of the manufacturing process of the CoDico plates for the CDP test with (**a**) the mold with 400 mm × 400 mm polished steel surface (**b**) the mold with inserted pre-consolidated UD tape with PTFE foil at one point for targeted crack initiation (**c**) with plastificate placed on the PTFE foil (**c**) with the plastificate placed on the PTFE foil (**d**) with another UD tape on top of the plastificate (**e**) after the pressing process, the mold filled with UD layer on top (**f**) 400 mm × 400 mm plate with drawn section (**g**) cross section of the plate with UD layer, LFT and PTFE foil for later crack initiation.

Production of UD tape, LFT-D based CoDico FRTP is a two-step process. Tape layups were produced on a Fiberforge made by Dieffenbacher GmbH Maschinen- und Anlagenbau, Eppingen, Germany. Layups comprise two overlapping layers of 0.13 mm thickness each and have dimensions slightly exceeding 700 mm by 700 mm accounting for overlap. Tapes were consolidated at 80 °C and 20 bar between two metal plates in a Dieffenbacher DYL 630 t hydraulic press after being heated to 280 °C in a contact oven made by WICKERT Maschinenbau GmbH, Landau in der Pfalz, Germany. Consolidated UD sheets are cut to 350 mm × 350 mm for co-molding. To facilitate the initial crack for interface testing, a polytetrafluorethylen (PTFE) foil was additionally fixed on the Co-phase before co-molding (Figure 4b). Time coordinated with compounding of Dico materials the UD sheets are heated to 280 °C in the contact oven between two layers of PTFE foil. Handling of the otherwise unstable UD layers is done solely with the PTFE foil to preserve fiber orientation. The order of production can be referenced in Figure 4. The first UD layer with PTFE foil for crack initialization is heated and placed in the mold (Figure 4b). PA 6 CF LFT-D material with a fiber mass (volume) fraction $w_{f}=34$ % ($v_{f}=26$%) is compounded at a rate of 39 kg/h, 280 °C barrel temperature, a screw speed of 59 rpm and a fiber roving count of eleven. The semi-finished material, called plastificate, is placed into the mold on top of the PTFE foil and first UD layer (Figure 4c). The second UD layer is placed on top of the plastificate and the sandwich structure is molded (Figure 4d,e). A fast press closing profile of 30 mm/s is chosen to minimize cooling of all constituents. The plates are 3 mm thick and molded at 200 bar for 35 s.

### 3.2. Specimen Preparation

The specimens were cut from the pressed plates in an iCUTwater smart of the company imes-icore GmbH, Eiterfeld, Germany, with a pressure of 1500 bar, a cutting speed of 200 mm/min and a flow rate of 250 g/min of cutting sand Classic Cut 120 garnet of the company GMA. The side with the initial crack through the PTFE foil was placed up in the waterjet system. The initial cut was made at the top of the sample since there is no PTFE foil avoiding delaminations.

After water jet cutting, the samples were dried in a vacuum oven at 50 °C for at least 240 h. Subsequent gravimetric measurements showed no further changes in weight. Consequently, this condition was considered to be completely dry. The samples to be tested in the “Dried” state were then stored in an airtight desiccator with silica gel. All further conditioning states were exposed to humidification from the dried state. Due to the temperature-dependence of water diffusion in polyamide 6, the moist and wet states were subjected to elevated temperatures (50 °C) to expedite the process. For the “Moist” condition, samples were placed in a climate chamber with 80% r.H., while for the “Wet” condition, samples were immersed in distilled water. After storing the samples for 240 h in their respective environments, gravimetric measurements indicated no further weight increase. Consequently, it is inferred that the saturation state has been attained in each environment. For the final condition, “RC” (i.e., room climate), the samples were placed in an air-conditioned laboratory with standard climate conditions. Due to the lower temperature in this environment, the diffusion processes required a longer duration. As a result, no significant weight change was observed after a longer period, up to 1500 h. The condition states were selected so that they represent the maximum and minimum moisture load as well as two intermediate states. Table 1 summarizes the various condensation processes. The samples were kept in their conditioning environment until testing to avoid further drying or wetting.

### 3.3. Parameters of Climbing Drum Peel Test

The CDP was tested on a zwickiLine universal testing machine from ZwickRoell AG, Ulm, Germany with a load cell with 2.5 kN capacity. For the drum a hollow aluminum cylinder with an (axially) outer radius of $r_{2}=62.5\;$mm and an (axially) center radius of $r_{1}=50\;$mm was used. To fix the specimen to the drum, a drum support was designed to hold the drum in the start position. At the beginning of the test, a preload of 90N was applied at a speed of 10mm/min. At 90N, the UD layer is partially rolled up on the drum, but no crack growth occurs. At the 90N preload, the actual test was started and the crosshead was moved upward at a speed of 100mm/min. After 80mm of travel, the test was stopped and the crosshead was returned to the start position at 50 mm/min, still recording the force.

### 3.4. Fractography

To examine the fracture surface after testing, the specimens were prepared in such a way that the UD tape was separated from the rest of the specimen up to the end of the crack. Pictures were taken with a DSLR camera with the fracture surface of both parts, i.e., Co and Dico, facing towards the camera.

Selected rectangles of 10 mm × 10 mm were cut from the Co tape within the fracture zone, which were subsequently sputtered with a platinum film with a thickness of 2 nm from both sides. These rectangles were investigated in a S-3400n SEM from Hitachi Ltd. Corporation, Chiyoda, Japan. The accelerating voltage was set to 10 kV and the working distance was kept around 14.5 mm. Since the SEM investigation took place over several days, the brightness and contrast was set individually, so that small variations in the picture quality are inevitable.

## 4. Numerical Model and Methods

### 4.1. Kinematics and Material Parameters

**Figure 5:**
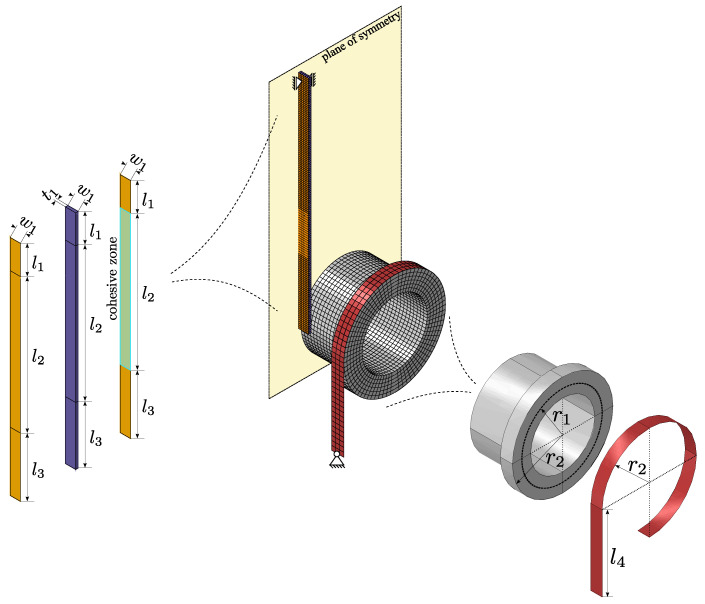
Assembly of the numerical model with the mesh structure of the final simulation and corresponding part geometries. The relevant dimensions (and purpose) in the model are $l_{1}=35\;$mm (clamping), $l_{2}=165\;$mm (cohesive layer), $l_{3}=72\;$mm (free end), $w_{1}=12.5\;$mm and $t_{1}=3\;$mm for the CoDico structure, and $r_{1}=50\;$mm (peel radius), $r_{2}=62.5\;$mm (winding radius) and $l_{4}=100\;$mm (free ribbon length) for the test rig dimensions.

In order to be able to use the qualitative characteristics of the experiments in simulations in which the delamination process must be taken into account, the experiment is to be simulated numerically in order to be able to make statements as to whether this is possible with the model presented below. For the simulation Abaqus FEA is used. Since the experiment is geometric non-linear with complex contact boundary conditions, an explicit time integration is chosen. The numerical model consists of four parts, all of which make use of the symmetry in the experiment. Therefore, only half of the experiment was simulated. The full assembly and the geometric properties are given in Figure 5.

The drum is modeled as a rigid body (R3D4) with a uniformly distributed mass density. Horizontal motion of its center is constrained, allowing only a vertical displacement. Likewise, all rotational degrees of freedom are constrained, with only the rotation about its central axis left unconstrained. The straps are modeled as an isotropic membrane structure (M3D4R) assuming the elastic properties of steel. Using membrane elements instead of shell elements has the advantage of avoiding bending moments. The upper part of the band is wrapped contiguously around the drum for 270° and the very end is attached to $r_{2}$ of the drum. The lower end of the band is fixed. The Dico structure is a 3D deformable model (C3D8R) with an anisotropic material assumption. The Co structure consists of shell elements (S4) with an orthotropic material symmetry. One Co part is attached coherently to the backside of the Dico part without the possibility of separating. Another Co part is attached to the front side of the Dico part using cohesive surfaces, leaving the lower part disconnected where the PTFE foil would be positioned in the experiment. Its lower end is connected to $r_{1}$ of the drum. The upper part of the specimen is partitioned and connected coherently to model the clamping of the specimen.

**Figure 6:**
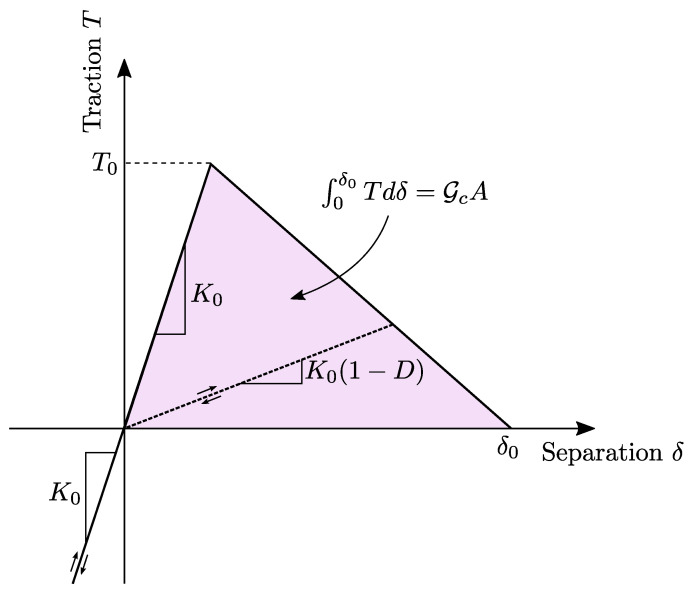
Schematic bi-linear traction-separation law in reference to [40], where $K_{0}$ is the intial stiffness, *D* is the damage variable, $T_{0}$ is the damage initiation traction, $\delta_{0}$ is the maximum separation, $G_{c}$ is the critical energy release rate and *A* is the fracture surface.

Essential for the numerical simulation of the interface effects in this study is the projection of the interface mechanics to cohesive surfaces. Inherent to cohesive formulations is that the crack can only develop along a predefined path, which in this case is limited to the interface between the Co and Dico structure. Cohesive surfaces relate the crack opening separation vector $\underline{\delta }$ at a given location, i.e., the displacement jump of adjacent continuum elements, to the traction vector $\underline{T}$ on this surface in the form of a traction-separation law

(6) $$\underline{T}=\underline{\underline{K}}\;\underline{\delta },$$

where $\underline{\underline{K}}$ is the stiffness matrix, which represents the resistance against a change in separation [38]. Incorporating the assumption of an identical stiffness in the normal and both shear directions and no shear coupling, the traction separation law simplifies to

(7) $$\underline{T}=K\underline{\delta },$$

with a single scalar stiffness *K*. Once the quadratic nominal stress damage initiation criteria is satisfied, which is

(8) $${\left(\frac{\langle T_{n}\rangle }{T_{0}}\right)}^{2}+{\left(\frac{T_{s}}{T_{0}}\right)}^{2}+{\left(\frac{T_{t}}{T_{0}}\right)}^{2}=1,\;\mathrm{where}\;\langle \cdot \rangle =\max\left(0,\cdot \right),$$

damage and a subsequent reduction in stiffness is modeled with an increasing damage variable D∈[0,1], such that the scalar stiffness reduces as $K=K_{0}\left(1-D\right)$ from an initial stiffness $K_{0}$. Here $T_{n}$, $T_{s}$ and $T_{t}$ are the normal and both shear tractions, respectively, while $T_{0}$ is the damage initiation traction. The development of the damage variable depends on the shape of the traction-separation law and reaches a maximum value of D=1, when a critical energy release rate $G_{c}$ is reached, indicating full separation. Further details on the mechanics of cohesive surfaces are given in [38,39]. In this study, the traction-separation law is chosen to be bi-linear for simplicity reasons, which is schematically depicted in Figure 6. Since $K_{0}$ is essentially a numerical value, which needs to be chosen high enough for numerical stability, two parameters, i.e., the critical traction $T_{0}$ and fracture energy G are free to be chosen to capture interface effects on the effective force-displacement curve in the simulation.

**Figure 7:**
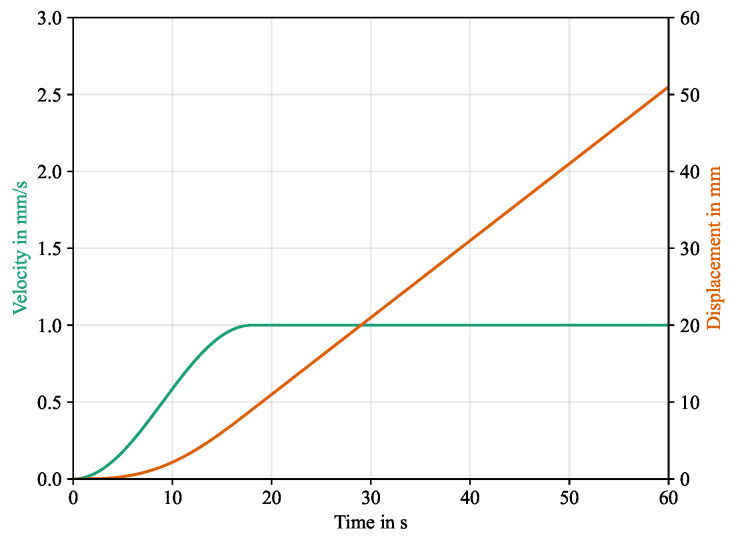
Displacement rate and corresponding displacement over time of the upper clamp in the simulation step.

The simulation consists of a single step in which a displacement rate is prescribed to the upper clamped part of the specimen. Preliminary simulations with the explicit solver revealed excessive vibrations in the model when the displacement rate was assumed to be constant over the whole step. To alleviate this, the displacement rate was applied in the initial phase with an increasing cosine function, as can be seen in Figure 7. Since gravitational forces also induced significant vibrations in the model, they were removed. Consequently, the average winding force $F_{w}$ is equal to zero in the simulation, representing an offset or shift to the experimental results. The separation energy in the interface is not affected by this, so that the results can be shifted to the experimental base line.

**Figure 8:**
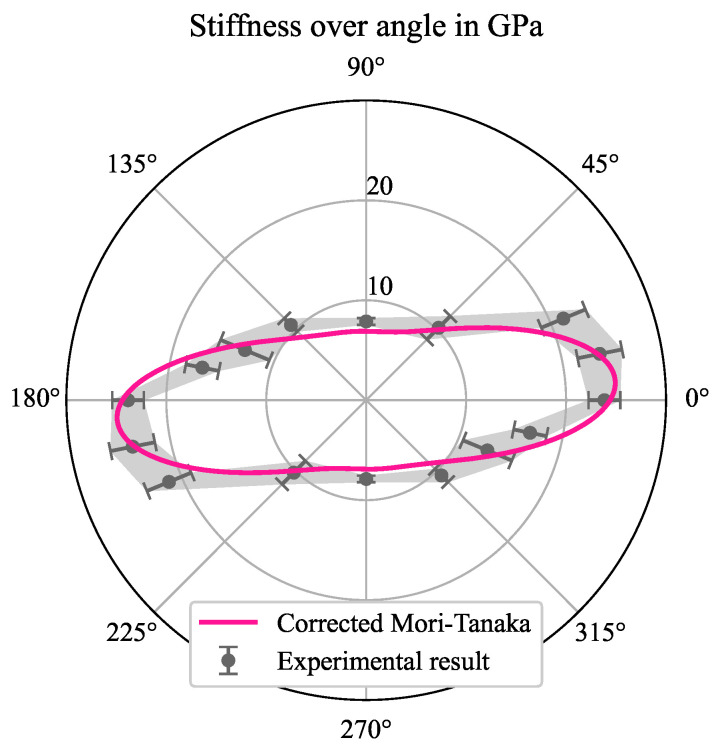
Polar plot of the stiffness properties of CF PA 6 (Dico) within the plane parallel to the casting mold: experimental results from Scheuring et al. (2024) and a corrected Mori-Tanaka homogenization using HomoPy (cf. [41]).

The linear elastic material parameters for both FRP materials were calculated by using a Mori-Tanaka (Dico) and a Halpin-Tsai homogenization procedure in combination with the laminate theory (Co), respectively, which are provided by the authors in the Python package HomoPy [41]. The input parameters for the homogenization methods were taken from a previous study under review on the same material system [42]. The findings in the mentioned study were that the stiffness properties of carbon long fiber reinforced PA 6 is overestimated when using a Mori-Tanaka homogenization. Reasons for this are that the experimentally determined aspect ratio is not enough to describe the complex microstructure including fiber interactions and bundle agglomerations, which were visible in µCT scans. As a result, the homogenization methods would overestimate the stiffness properties in the longitudinal direction. To circumvent this, a synthetic aspect ratio in alignment with [43] was used to fit the longitudinal stiffness properties to the experimental results in Scheuring et al. (2024). Corrected results are compared to experimental findings in Figure 8. All material parameters are listed in Table 2 and Table 3. The authors would like to mention that the material parameters were not changed in respect to humidity effects. Only the parameters within the cohesive surface modeling were altered.

Simulations were performed with a general contact formulation, with the exception of the cohesive surface definition between the Co and Dico structures.

### 4.2. General Numerical Studies

To evaluate the sensitivity of the model a study for the following properties was performed:

- mesh sensitivity,
- mass scaling,
- material properties of Dico and Co,
- cohesive parameters.

#### 4.2.1. Mesh Sensitivity

To determine a suitable mesh density for the Co and Dico layer in the simulation, a mesh study is performed while the material parameters are kept constant. The mesh properties of the other parts (drum and band) are unaltered. Three different configurations are tested with the given material properties in Table 2 and Table 3, a constant mass scaling factor of 64 and the following cohesive surface parameters:

*Damage Initiation, criterion=QUADS

1.,1.,1.

*Damage Evolution, type=ENERGY

1.071,

*Damage Stabilization

0.0001

The coarse configuration consists of elements with the dimensions 8.00mm×6.25mm for the shell elements (Co) and 8.00mm×6.25mm×3.00mm for the continuum elements (Dico), giving a total of 3777 elements in the simulation. The element dimensions for the medium case are chosen with $4.00\;\mathrm{mm}\times 4.1\bar{6}\;\mathrm{mm}$ and $4.00\;\mathrm{mm}\times 4.1\bar{6}\;\mathrm{mm}\times 3.00\;\mathrm{mm}$, respectively, giving a total of 4185 elements. For the fine case the element dimensions are given with $2.00\;\mathrm{mm}\times 2.08\bar{3}\;\mathrm{mm}$ and $2.00\;\mathrm{mm}\times 2.08\bar{3}\;\mathrm{mm}\times 1.5\;\mathrm{mm}$, respectively, giving a total of 6861 elements. Simulation times are recorded and a force-displacement curve is generated for each case and compared qualitatively.

#### 4.2.2. Mass Scaling

Since it is always advantageous to shorten simulation run times, it seems obvious to increase the mass scaling (MS) factor in explicit simulations in order to increase the critical time increment. It is important to consider the ratio between internal and kinetic energy to avoid a significant influence of inertial effects in a quasi-static experiment and consequently to keep unwanted oscillations within the model due to excessive mass scaling in check. To find a suitable mass scaling factor, different mass scale factors are tested for otherwise constant simulation parameters. For this, mass scaling factors are chosen in the following set [1,4,16,64,256], where a mass scaling factor of 1 implies no mass scaling after all. The resulting energies and run times are compared.

#### 4.2.3. Material Properties of Dico and Co

Since to the best of the authors’ knowledge the CDP experiment has not been evaluated in a full 3D simulation before, it is of interest to investigate whether and to what extend the material properties of the Dico and Co models affect the force-displacement results. For simplicity reasons, the material properties are assumed to be linearly elastic and isotropic. The cohesive parameters are kept constant as given before. Since an extensive study on this topic to cover many material parameter combination is out of scope of this research, only a few combinations are tested. Therefore, the Poisson’s ratio is kept constant for both materials at ν=0.3 and the densities from Table 2, while choosing the Young’s moduli as in Table 4. A reference stiffness of 110GPa and 25GPa is chosen for Co and Dico, respectively. The stiffness values are doubled and combined in all possible ways, giving four different combinations. The force-displacement curves will be compared. To avoid the effects of the cohesive zone parameters, the simulation will also be run for a disabled cohesive interaction.

#### 4.2.4. Cohesive Parameters

Lastly, a preliminary study on the effects of the cohesive parameters in the bi-linear cohesive surface approach in Abaqus are performed. The most important parameters are the damage initiation traction/stress value and the critical energy release rate. The first parameter sets the traction at which damage first occurs within a cohesive surface, while the second parameter determines the corresponding damage evolution for a further increase in traction. Once the displacement energy per fracture area reaches the critical energy release rate, the interface in this specific element is assumed to be fully delaminated, providing no more resistance. While the effects of parameter variation are obvious for a single element, the interpretation for a multi element simulation with complex boundary conditions is not trivial. Hence, a study will be performed to investigate these effects for the given model. Since this is a cursory study, combination is limited to a discrete parameter space of dimensions 2×3 in energy and traction. Preliminary investigations revealed that a damage initiation value above 10N/mm^2^ leads to implausibly large magnitudes of oscillations, which gives an upper bound. The exact combinations are given in Table 5.

#### 4.2.5. Full Simulation of Experiment

**Figure 9:**
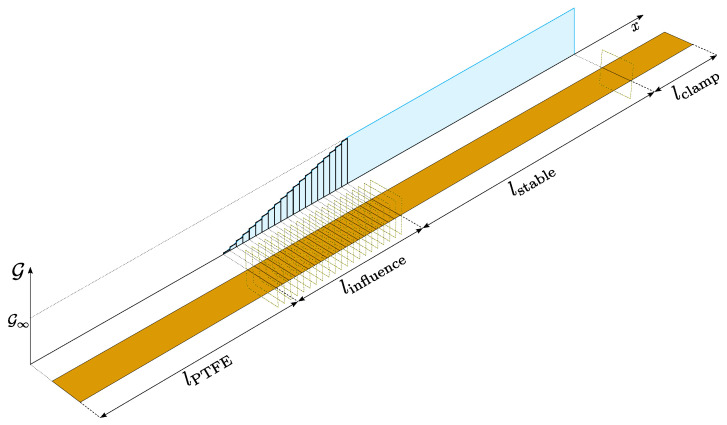
Distribution of the critical energy release rate within the cohesive zones for the Co tape. The cohesive length is split into a stable length, where the energy is assumed to be constant, and an influence length, in which the energy increases for each segment by a constant step to approximate the assumption of a linear distribution between the PTFE foil and the stable region.

Following the numerical studies, certain modifications had to be made to the final simulation approach in order to capture the effects of the measured experiments as accurately as possible with the means introduced, which are explained here in anticipation. In particular, the experimental results show that there is a zone of influence in which the interface parameters have not reached their full potential. Reasons for this are explained in Section 5. Assuming that the interface has constant parameters over its entire length, a sudden increase in force could be observed in the simulation, as opposed to a smooth linear increase over a certain length, the so-called zone of influence, as seen in the experiment. To capture this effect, the interface properties are assumed to be piecewise constant in segments over the influence zone with a segment number of n=20 with a total length of 48mm, where the edge to the PTFE foil has a critical energy release rate close to zero and increases with a constant step towards the other end of the influence zone until the final value of the fracture energy is reached. This is illustrated in Figure 9.

## 5. Results and Discussion

### 5.1. Experimental Results

Before the experiments were performed, the specimens were weighed to calculate their water uptake in mass percent. These results are given in Table 6. It can be seen that an increase of humidity from the room climate (RC) conditioning to a moist conditioning doubled the moisture content, while the immersion in water again more than tripled the water content in comparison to the moist conditioning.

**Figure 10:**
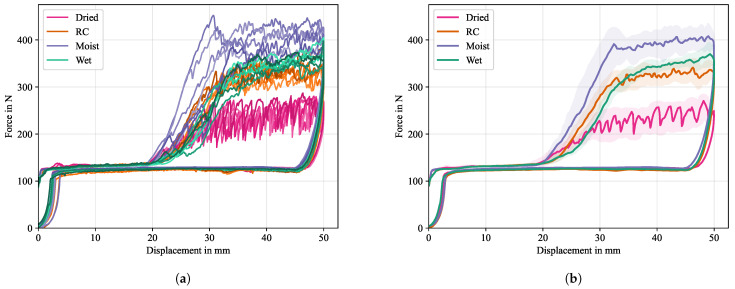
Experimental results of CDP test. (**a**) shows the individual results and (**b**) is the averaged result for each conditioning class using an arc-length based averaging scheme (cf. [44]) where the envelopes indicate statistical response corridors.

Experimental force-displacement curves are given in Figure 10a, while the averaged result is given in Figure 10b. Here, the arc-length parametrization introduced in [44] was used, which has the advantage of preserving certain characteristics in the force-displacement signal, e.g., magnitude and position of oscillations, and the ability to work on hysteretic data, as is the case for the loading-unloading experiment.

Based on the kinematics of the test set-up, the initial rise of the force signal ends after a specimen displacement of about 2 mm, once the drum is fully lifted, at a constant $F_{w}$ of 120 N. The force signal is unaltered until a specimen displacement of about 20 mm is reached, indicating a crack position right at the end of the PTFE foil. After the PTFE foil, the CoDico interface is yet fully intact, requiring an increase in the force signal to propagate the crack. All conditioning states show an initial incline zone, where the force has not reached a stable value. Reasons for this are boundary effects from the cooled down plastificate in the vicinity of the PTFE inlay, which are also visible in the later discussed optical analysis in Section 5.1.1. After about 32 mm of displacement a maximum, stable (on average) force level is reached with periodic force oscillations. These oscillations are caused by a stick-slip crack propagation, indicating that the crack front is not on par with the exact position of the center of the drum.

Significant differences in the force signal between the four conditioning states are evident. Dried specimens exhibit the lowest maximum force with an average $F_{d}$ of about 220 N, but have the strongest fluctuations in force signal, indicating pronounced erratic crack propagation, which could also be heard during the experiment. As moisture content increases, the maximum force initially increases, i.e., a $F_{d}$ of about 320N for RC and 400 N for moist specimens, with fluctuations increasingly weakening. For the specimens immersed in water, however, the maximum force drops again to a lower value slightly above the RC value at around 340 N.

**Figure 11:**
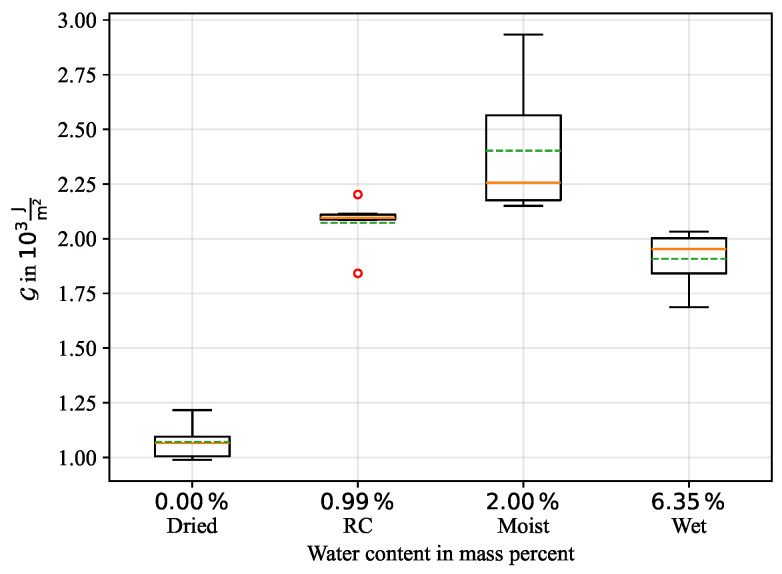
Box plot of the critical energy release rate $G_{c}$ for all conditioning states.

When the maximum displacement of 50 mm is reached the traverse reverts to its initial position, decreasing the force signal slightly below the initial $F_{w}$ value. Based on the procedure indicated in Figure 2, the enveloped area of the force-displacement diagram was determined numerically for all curves. The newly formed fracture area was measured, so that the critical energy release rate $G_{c}$, or CERR, could be calculated by using Equation (5). A statistical box plot for all conditioning states is given in Figure 11. It can be seen that the lowest mean CERR was achieved by the dried samples with $G_{c}=1.07\cdot 10^{3}\;J/m^{2}$, followed by RC with $G_{c}=2.07\cdot 10^{3}\;J/m^{2}$ and the moist conditioning with $G_{c}=2.40\cdot 10^{3}\;J/m^{2}$, indicating a better energy absorption for an increase in moisture. While the variation in experimental results is low for the dried and RC conditioning, an increased variation is observed for the moist conditioning. Contrary to the upward trend in CERR with increasing moisture content, a further increase of humidity in the case of the immersed samples weakens CERR with $G_{c}=1.91\cdot 10^{3}\;J/m^{2}$. Possible reasons for this are discussed in the following sections.

#### 5.1.1. Fractography

**Figure 12:**
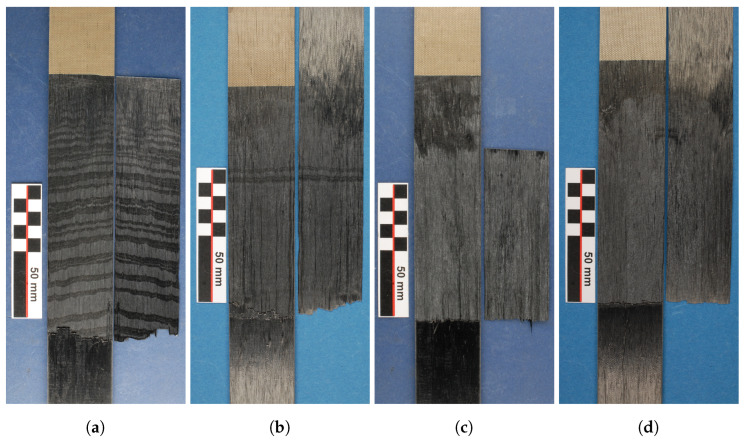
Fractography results of CDP test. (**a**) shows the fracture surface of the dried specimen with a clear stick-slip (alternating ductile/brittle) fracture pattern (parts of the Co tape were cut away for investigation), (**b**) shows the fracture surface of the specimen conditioned at room climate with minor signs of a stick-slip effect. (**c**,**d**) show the fracture surface of the moist and fully immersed specimens, respectively. All specimens show an altered initial zone in the vicinity of the PTFE foil.

The images of the fracture surfaces are displayed in Figure 12. A clear distinction between the four different conditioning states can be observed in the fracture surfaces. Most dominantly, the fracture surface of the dried specimen (cf. Figure 12a) shows an alternating fracture pattern with bright and dark regions, which repeats itself through all specimens. Here, the bright regions increase in size for an increased crack propagation (away from the PTFE foil). The number of oscillations correlate with the force drops in the force-displacement curve for dried specimens. This pattern is also visible in some of the fracture surfaces of the conditioned specimens at room temperature in isolated regions, as can be seen in Figure 12b. For elevated humidity levels (cf. moist and wet), the fracture surface does not show this pattern but instead consists of a homogeneous bright area. For all conditioning states, the first few centimeters after the PTFE foil reveal an altered fracture surface with a darker tint, indicating an initial influence zone in the vicinity of the PTFE foil. The length of the influence zone in the vicinity of the PTFE foil correlates with the region in the force-displacement diagram before a stable force value $F_{d}$ is reached, causing the force to steadily increases instead of suddenly reaching a stable value (cf. Figure 10). The interpretation is that in the vicinity of the PTFE foil a weaker consolidation occurs during the molding process due to the positioning of the LFT-D plastificate (cf. Figure 4). Since the outer surface of the plastificate already cooled down, a weaker consolidation is achieved between the Dico plastificate and the Co tapes. Once the plastificate fills the mold, still warmer material from the inside of the plastificate gets into contact with the areas further away from the PTFE foil, creating a better bond. Thus less energy is required in the area close to the plastificate position to propagate the crack, which explains the gradual increase in force further away from the PTFE foil until a uniform consolidation quality and consequently a stable energy rate is achieved.

**Figure 13:**
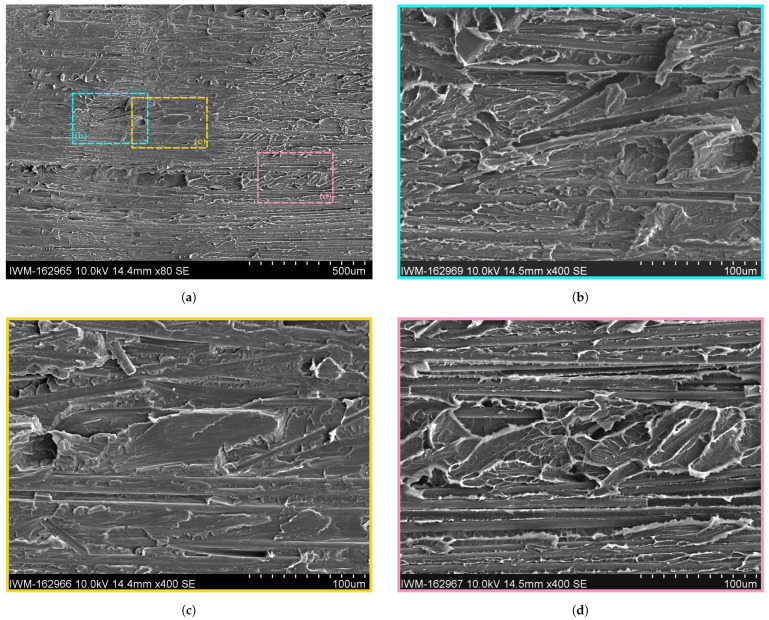
SEM images of fracture surface of dried sample with details of brittle and ductile fracture zones on the Co side. (**a**) SEM image of fracture surface of dried sample at ×80 magnification. (**b**) Detailed SEM image of fracture surface of dried sample of brittle-ductile transition zone at ×400 magnification. (**c**) Detailed SEM image of fracture surface of dried sample of brittle zone at ×400 magnification. (**d**) Detailed SEM image of fracture surface of dried sample of ductile zone at ×400 magnification.

Taking a closer look at the alternation pattern in the SEM in Figure 13 shows a good visibility of the alternating fracture pattern. It becomes clear that the alternating pattern is a repetitive switch between a brittle and ductile crack propagation. In the SEM, the ductile fracture zone (cf. the magenta box or Figure 13d) is characterized by diffuse, fringed edges caused by excessive plastic deformation of the matrix material. Next to the polymer fringes, many fibers from the Co FRTP are visible. The upper sides of the fibers towards the detector are mostly blank with minor polymer residues, indicating a crack propagation along the fiber-matrix interface of the Co tape. The ductile areas in the fracture zone correlate with the bright sections in the images in Figure 12. This can be explained by a high degree of light dispersion due to the rough surface caused by the polymer fringes, so that light entering from the sides directs more light onto the camera’s sensor and creates a brighter surface. Consequently, the only bright fracture surfaces for the moist and wet specimens must be ductile. 

The brittle fracture zone in the yellow box or Figure 13c is characterized by a flat and dull surface with slightly elevated plateaus with sharp edges. Contrary to the ductile fracture zone, blank fiber surfaces are not visible. Instead, fiber groves are visible, indicating that the fibers of the Co tape stuck to the Dico side when the crack propagated.

In the transitioning zone (cf. Figure 13b) it can be seen that the transition from ductile to brittle fracture occurs gradually, but over a short distance of a few microns. The fringes decrease in size until the flat and dull characteristics of the brittle zone are reached.

**Figure 14:**
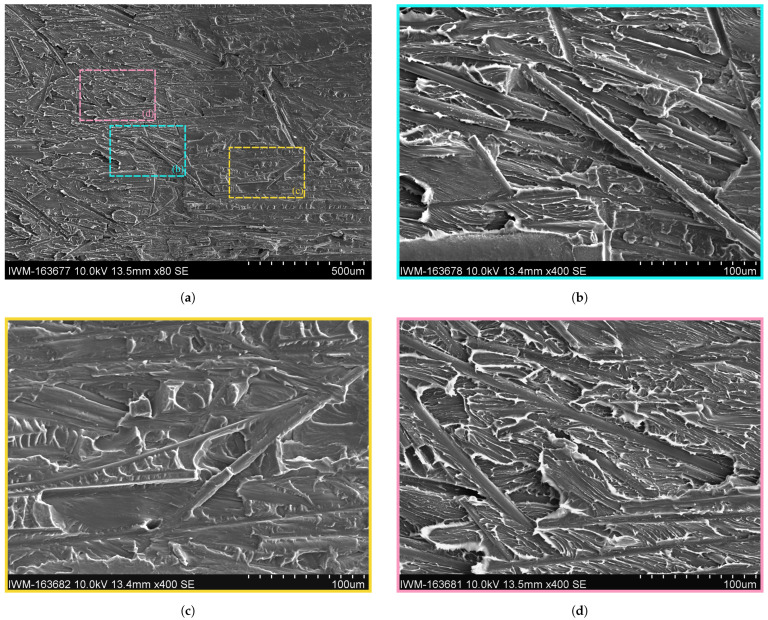
SEM images of fracture surface of dried sample with details of brittle and ductile fracture zones on the Dico side. (**a**) SEM image of fracture surface of dried sample at ×80 magnification. (**b**) Detailed SEM image of fracture surface of dried sample of brittle-ductile transition zone at ×400 magnification. (**c**) Detailed SEM image of fracture surface of dried sample of brittle zone at ×400 magnification. (**d**) Detailed SEM image of fracture surface of dried sample of ductile zone at ×400 magnification.

A direct comparison with the complementary fracture surface of the Dico face is made in Figure 14. Similarly, to the discussed fracture surface on the Co side, a clear distinction between a brittle and ductile fracture zone is visible in the Dico side, indicated by diffuse, fringed edges for the ductile zone and a flat and dull surface for the brittle zone, respectively. Again, grooves of pulled out fibers are visible throughout the fracture zone. The remaining fibers in the brittle fracture zone seem to be surrounded by matrix material, indicating a good interface bonding (cf. Figure 14c). The fibers in the ductile fracture zone appear to have suffered more interface damage, while some of the matrix material remains on the fiber surface (cf. Figure 14d).

**Figure 15:**
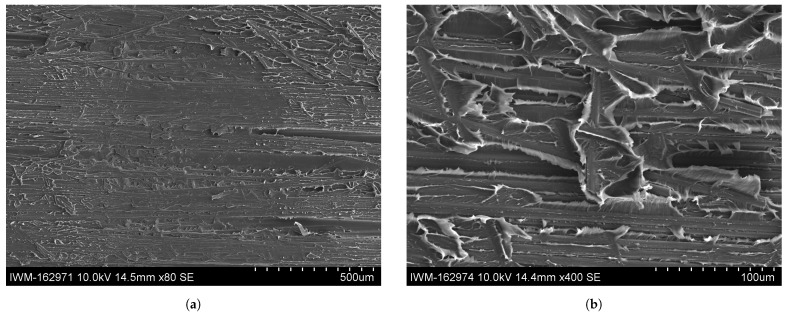
SEM images of fracture surface of RC samples on the Co side. (**a**) SEM image of fracture surface of RC sample at ×80 magnification. (**b**) Detailed SEM image of fracture surface of RC sample at ×400 magnification.

**Figure 16:**
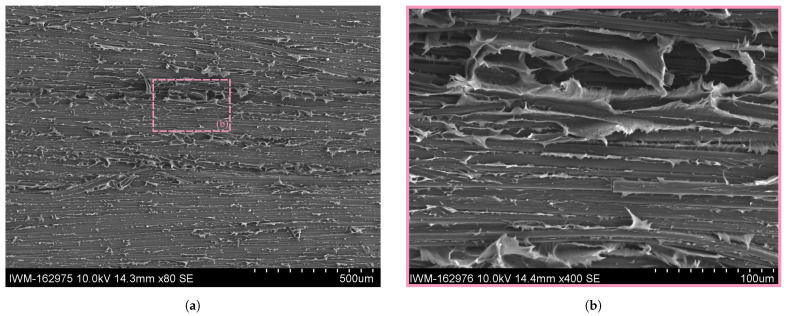
SEM images of fracture surface of moist samples on the Co side. (**a**) SEM image of fracture surface of moist sample at ×80 magnification. (**b**) Detailed SEM image of fracture surface of moist sample at ×400 magnification.

**Figure 17:**
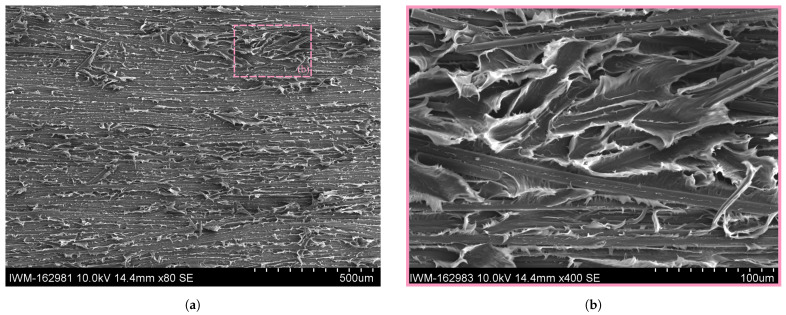
SEM images of fracture surface of wet samples on the Co side. (**a**) SEM image of fracture surface of wet sample at ×80 magnification. (**b**) Detailed SEM image of fracture surface of wet sample at ×400 magnification.

A comparison with the ductile fracture pattern within the Co tape in the RC, moist and wet specimens is shown in Figure 15, Figure 16 and Figure 17. Apart from the aforementioned finding that the specimens conditioned at room climate occasionally show brittle fracture zones (cf. Figure 15a), no systematic deviation in the fracture pattern within the ductile zones could be found. All conditioning states show continuous fibers with a debonded interface, with polymer residues dispersed along the fiber length. It can be seen that the connecting matrix material between two adjacent fibers shows pronounced plastic deformation in the form of polymer fringes or wrinkles.

Alternation between ductile and brittle fracture pattern is the reason for pronounced stick-slip crack propagation for dried specimens and for the large force oscillations. Brittle fracture is only rarely seen for specimens conditioned at room temperature and never for moist and wet cases. The explanation is that water absorption within PA 6 leads to a plasticization of the polymer, enhancing the chain mobility within the thermoplastic. Thus, plastic deformation is enhanced. Owing to the more brittle material behavior in dried specimens, crack propagation is hindered and an offset occurs between the crack tip and drum position. This offset increases normal stresses near the crack tip, which, at a critical value causes unstable crack growth so that the crack catches up with the current drum position, preventing crack propagation again. When the drum advances further, stable crack growth initially occurs until the difference between the positions is again large enough to cause a critical stress value and thus unstable crack growth. This behavior explains the stick-slip behavior and the alternation pattern in the fracture surface. In case of moisture plasticized polymer, such stress peaks can be relieved by plastic deformation, resulting in gradual stable crack growth and no stick-slip behavior. Owing to higher deformation energy in highly plastically deformed polymer, the fracture work performed increases, which is confirmed in experiments as moisture content increases. Exceptions are specimens with maximum moisture, which were immersed in water.

One possible cause of this could be hydrolytic processes that take place in contact with water at temperatures above $T_{g}$. During the hydrolysis process, water reactions break amide groups in polyamide molecules, which leads to a gradual degradation of the polymer structure. This leads to a reduction in molecular mass and viscosity, which has a negative effect on mechanical properties such as tensile strength and hardness. Since $T_{g}$ is decreasing due to absorbed water and the aging has been going on for a longer time at 50 °C, it can be assumed that such effects are occurring. To verify this, it would be useful to store samples in water at lower temperatures and compare the results. There also is a potential for hydrolysis occurring at the interface between the fibers and the matrix. Reinforcing fibers are commonly treated with sizing to enhance this interface, typically composed of silane or epoxy resin. However, it’s worth noting that both silane and epoxy resin are susceptible to hydrolysis.

### 5.2. Numerical Results

#### 5.2.1. General Numerical Studies

**Figure 18:**
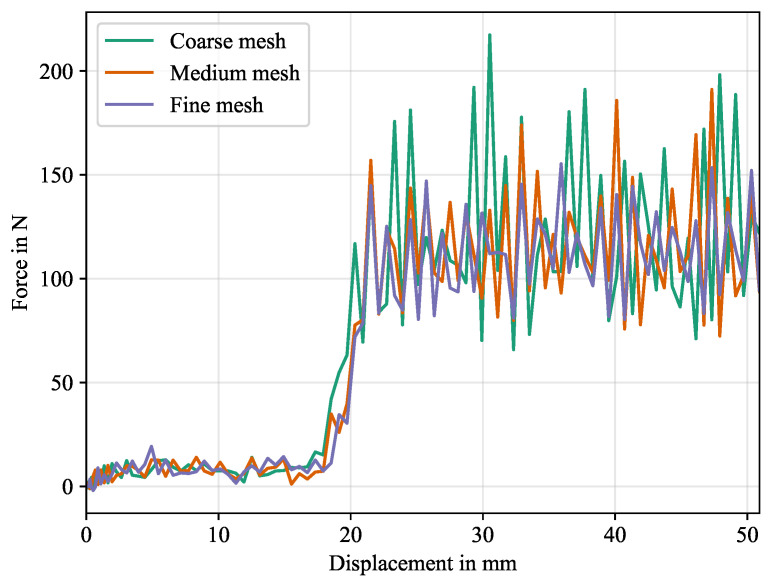
Force-displacement curves for mesh study. The force signals were multiplied by 2 to account for the symmetry condition.

The first generic study was the mesh sensitivity study for which the recorded simulation times are given in Table 7. While the medium mesh density takes less than twice the computational effort of the coarse mesh, the fine mesh takes almost eight times as much run time to complete. Force-displacement plots are given in Figure 18.

Oscillations magnitudes decrease for a finer mesh. Specifically the coarse mesh overestimates these magnitudes significantly in contrast to the fine mesh, while the results of the medium mesh density are close to the fine mesh, indicating a mesh convergence. Qualitatively, the initial force rise shortly before t=30s is matched well between medium and fine mesh. The results for coarse mesh differ here. Considering run time increasing by a factor of 4 from medium to fine mesh, a medium mesh density is deemed sufficient for the following studies.

#### 5.2.2. Mass Scaling

**Figure 19:**
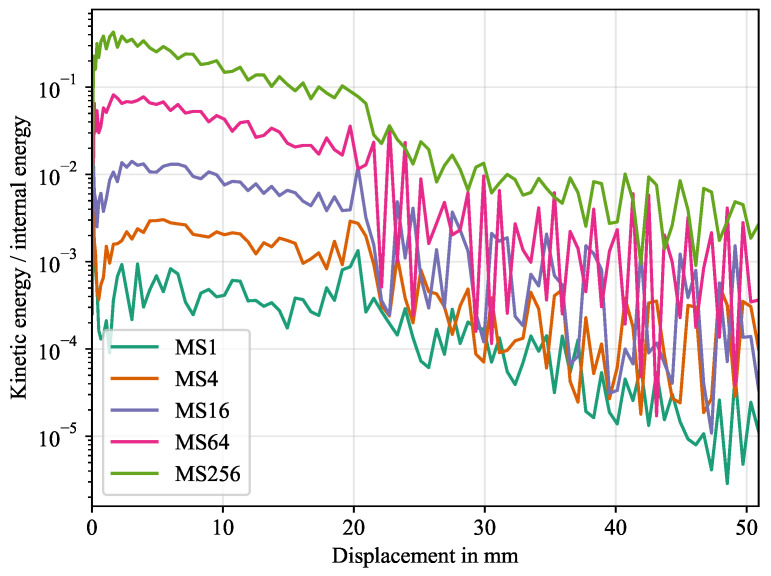
Ratio of kinetic energy to internal energy for different MS factors over displacement.

To find a suitable mass scaling factor, the ratio between kinetic energy per internal energy was evaluated in Figure 19. It can be seen that the ratio reduces up to a factor of almost ten for a reduction of mass scaling for a factor of four. Interestingly, a mass scaling factor of 64 results in a great magnitude of oscillation in the interface separation section, which is not seen for the other cases. The simulation with a mass scaling factor of 256 took roughly 10,000 s and the run time is about doubled for each reduction by a factor of 4, with a maximum run time of 160,000 s for a mass scaling factor 1. The exact run times are given in Table 8. To avoid large oscillations, while still reducing the run time, a mass scaling of 16 was chosen for the final simulations.

#### 5.2.3. Material Properties of Co and Dico

**Figure 20:**
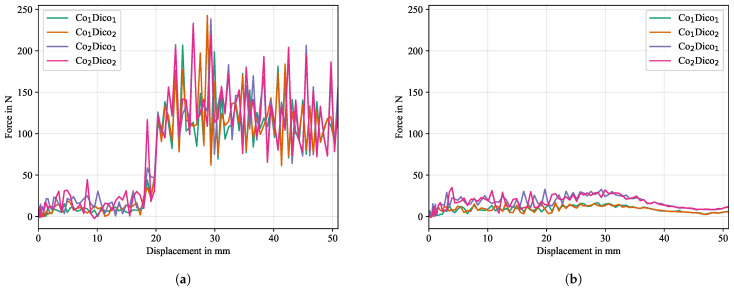
Force-displacement curves for the numerical study on the effects of material properties of Co and Dico: (**a**) with and (**b**) without cohesive effects.

Resulting force-displacement curves for different combinations of material properties for Co and Dico (cf. Table 4) are given in Figure 20, where Figure 20a represents the simulation with cohesive surfaces intact and Figure 20b represents the simulation without cohesive effects (no crack propagation, i.e., initially completely detached interface). From Figure 20b, i.e., simulation results without cohesive effects, numerical oscillations can be seen which vary in magnitude depending on the material combination. Since no gravitational effects are considered, the oscillations must result from the reaction forces from the bending resistance of the deformed Co tape and subsequently from the induced vibrations in the explicit model. The force-displacment curves can be separated in two groups. The first group consists of cases in which the Co FRTP stiffness is unchanged at 25GPa, while the other group is formed by the remaining complement with a Co FRTP stiffness of 50GPa. The first group shows magnitudes of less than half the magnitudes of the second group, while these magnitudes do not vary significantly within each group, indicating that the stiffness properties of the bulk material (i.e., Dico) from which the flexible structure (i.e., Co) is ought to be peeled off are of secondary importance for the resulting reaction forces. Consequently, while it is important to model all material properties as accurately as possible and necessary, the primary focus should be on modeling the properties of the flexible Co-layer, as the CDP test simulation is particularly sensitive to these parameters. The same picture emerges when cohesive effects are considered, as is the case for Figure 20a. Here, the magnitudes of oscillations in the interface separation section (>20 mm) is again significantly increased for a stiffer Co material, reinforcing the recommendation to be careful when modeling the flexible structure. The sensitivity on the chosen Co parameters may be reduced for a lower mass scaling factor (here a constant factor of 64 was used), which should be investigated in future studies.

#### 5.2.4. Cohesive Parameters

**Figure 21:**
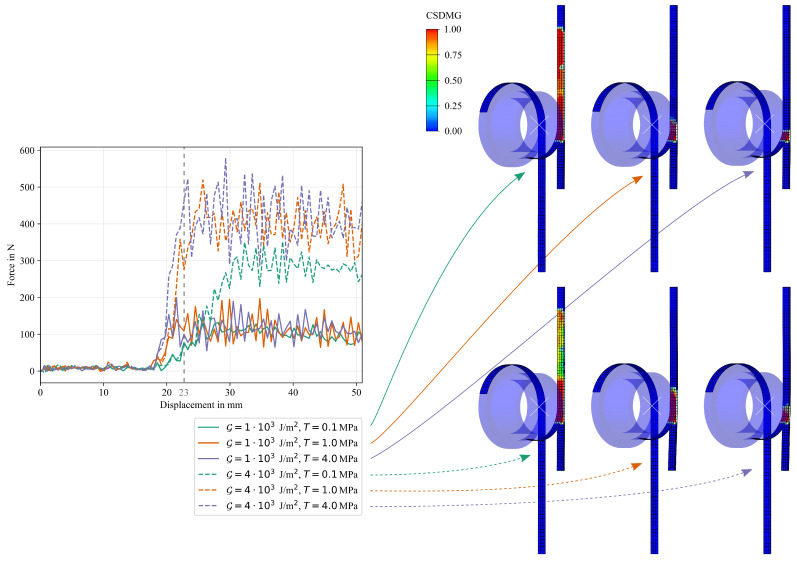
Force-displacement curves for the numerical study on the effects of cohesive parameters (left) and deformation plot with the CSDMG (overall value of the scalar damage variable) variable as contour at u=23mm for all six configurations. The main finding is that for a low traction value in the cohesive formulation, the interface damage (crack tip) advances the drum position excessively.

The results for the numerical study on the influence of cohesive parameters are given in Figure 21. It can be clearly seen that, in general, an increase in fracture toughness in the cohesive formulation results in an increase in the work required to propagate the crack, while an increase in damage initiation traction/stress results in slightly increased oscillations in the measured reaction force and a steeper increase at initial debonding without changing the area under the force-displacement curve. If the damage initiation traction/stress value is high enough, an increase in fracture toughness linearly increases the measured work. It is of interest that if the damage initiation value is too low, the work required to propagate the crack is reduced, which is evident in the curve for the combination of $G=4\cdot 10^{3}\;$J/m^2^ and $T_{0}=0.1\;$MPa. The reason for this can be explained by looking at the damage propagation at a displacement of u=23mm on the right side of Figure 21. It can be seen that for both fracture toughness values, the lowest damage initiation value of $T_{0}=0.1\;$MPa results in an advancement of the crack tip relative to the drum position, separating the Co tape from the Dico bulk material at an early stage of the simulation. Reasons could be either oscillations in the explicit simulations leading to stress peaks that advance the damage initiation value, or early failure in the shear mode due to in-plane stresses in the interface as the Co tape is pulled down.

During testing, the crack propagation is at the same level as the drum position, necessitating the selection of a sufficiently high value for the damage initiation parameter to prevent the crack from running ahead of the drum position. The numerical results show that a higher damage initiation value means that the crack tip is coherent with the drum position, which is confirmed by comparing the damage initiation stresses of 1.0MPa and 4.0MPa. Although the shape of the force-displacement curve at low damage initiation values appears to be more in agreement with the experimental result (cf. Figure 10), which is due to the structure consisting of an influence zone with reduced interfacial toughness leading to a smooth increase of the reaction force over a certain length, the reason is not accelerated crack propagation. Therefore, this effect must not be modeled by artificially reducing the damage initiation value.

#### 5.2.5. Final Simulation

**Figure 22:**
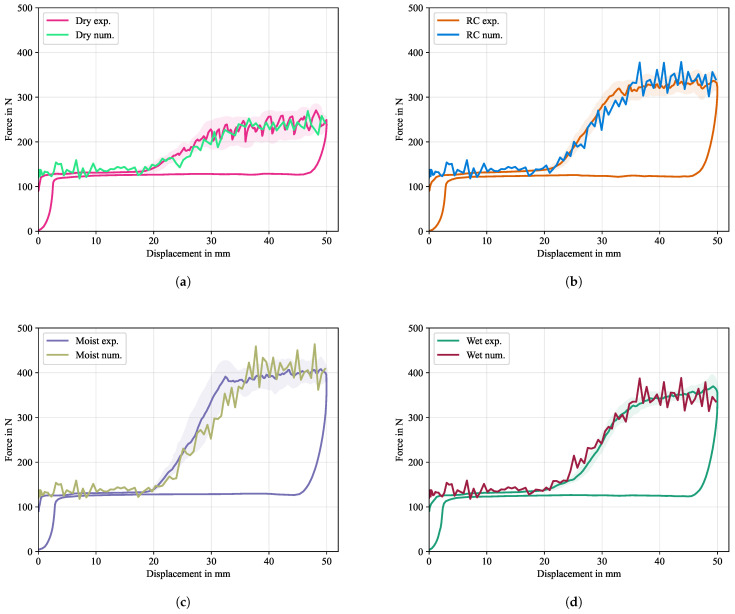
Numerical results of CDP test in comparison with the averaged experimental results and calculated response corridors using the arc-length based averaging scheme (cf. [44]). (**a**) shows the results for dried specimens, (**b**) for specimens conditioned at room climate, (**c**) moist specimens and (**d**) specimens immersed in water. The force signal was multiplied by 2 to account for the symmetric boundary condition and an offset of 129N was added to account for gravitational forces, which were not considered in the simulation.

Based on the preliminary studies, the final simulations were run with a given set of cohesive parameters, while leaving the material properties of the Co tape and the Dico bulk material unchanged. In the previous studies it could be observed, that a single cohesive property assignment over the whole interface leads to a sudden jump in the force-displacement signal (cf. Figure 18 and Figure 20a), which is in contrast to the gradual incline as seen in the experimental results (cf. Figure 10b) between the displacement values of d=20mm and d=32mm. Consequently, the approach of a piecewise constant assignment over 21 segments was chosen, as illustrated in Figure 9. The final value of the fracture energy for each conditioning scenario was calculated only from the stable section between a displacement of 32mm and 50mm from the averaged curves to not include the initial incline zone, using the method depicted in Figure 3 and Equation (4), respectively, with $\Delta u_{0}=18\;$mm. The CERR were determined to be $G=1.0\cdot 10^{3}\;$J/m^2^ for dried samples, $G=2.0\cdot 10^{3}\;$J/m^2^ for samples at room climate, $G=2.7\cdot 10^{3}\;$J/m^2^ for moist samples and $G=2.07\cdot 10^{3}\;$J/m^2^ for immersed samples, while the damage initiation stress was set to $T_{0}=1.0\;$MPa for all cases. Results of the final simulations are given in Figure 22.

In the initial force signal increased oscillations are observed that cannot be seen during the experiments. The reason for this is that at the beginning of the explicit simulation, oscillations build up that are not damped due to the lack of viscous properties in the material model. During interface formation, the oscillations continue with a slightly increased amplitude. The maximum separation force and energetic properties of the experiments are well captured for all conditioning states, indicating a high sensitivity to the cohesive parameter, especially to the separation energy. Only the moist condition simulation shows a slightly reduced rate of increase in the force signal (cf. Figure 22c), which results in a later average stabilization of the force signal. A necessary requirement of the given modeling approach was to divide the influence zone after the PTFE foil into smaller segments with piecewise constant cohesive properties in order to achieve a steady slope of the force signal, as could be seen in the experiments. When the drum reaches the last segment, the force signal remains constant in an average sense. In general, it needs to be pointed out that the chosen approach of using cohesive surfaces without having a model for processes in the microstructure (e.g., humidity controlled plasticization) can only capture the overall shape of the force-displacement curves, while concurring oscillations, as it is the case in Figure 22a cannot be explained by the model. To enhance the modeling capabilities and deepen the understanding of aforementioned processes in the microstructure, such as the alternating ductile/brittle crack propagation or hydrolitic effects, a more complicated material model would need to be developed.

## 6. Conclusions

This paper investigates the debonding behavior at the interface of continuous and discontinuous fiber reinforced thermoplastics using the climbing drum peel test. The moisture content of the specimens significantly affected the fracture behavior and the energy required for material separation. A balance between ductile and brittle fracture was observed in dried specimens, while specimens with a higher moisture content exhibited a purely ductile failure. The increase in fracture surface energy with moisture content was attributed to the plasticization of the polymer, enhancing polymer chain mobility and deformation within the fracture zone. However, further moisture increase led to a reduction in required energy due to hydrolitic effects and expected polymer degradation. Numerical modeling with cohesive surfaces was also performed for the first time, highlighting the sensitivity of the material parameters of the continuous reinforcement and the influence of damage initiation parameter and fracture surface energy on the effective force-displacement curve. The model successfully replicated the experimental force-displacement curve, demonstrating its potential for future research.

## Figures and Tables

**Table 1:** Conditioning procedure of the different conditioning states.

Label	Dried	RC	Moist	Wet
Conditioning	50 °C 0% r.H. in vacuum	23 °C 45% r.H.	50 °C 80% r.H.	50 °C submersed in distilled water
Duration	≥240 h	1500 h	240 h	240 h

**Table 2:** Material parameters used in the simulation. E_*i*_ and G_*ij*_ are given in MPa and ϱ is given in g/cm^3^, respectively.

	E_1_	E_2_	*ν*_12_	G_12_	G_13_	G_23_	ϱ
Drum	-	-	-	-	-	-	2.70
Bands	210,000.0	-	0.3	-	-	-	7.85
Co	108,700.0	11,039.2	0.3	4092.0	4092.0	1118.3	1.48
Dico	see Table 3						1.27

**Table 3:** Material parameters used for Dico in the simulation in MPa.

D_**1111**_	D_**1122**_	D_**2222**_	D_**1133**_	D_**2233**_	D_**3333**_	D_**1112**_
29480.2	6673.9	10412.1	4866.4	4983.5	8362.6	1.2
D_**2212**_	D_**3312**_	D_**1212**_	D_**1113**_	D_**2213**_	D_**3313**_	D_**1213**_
51.7	13.9	1736.2	−28.5	−3.0	11.5	8.7
D_**1313**_	D_**1123**_	D_**2223**_	D_**3323**_	D_**1223**_	D_**1323**_	D_**2323**_
1840.5	1170.9	142.6	−7.3	−2.7	0.6	3626.0

**Table 4:** Young’s moduli combination for Co and Dico to study the effects of material parameters on the force-displacement curve.

Label	Co_**1**_Dico_**1**_	Co_**2**_Dico_**1**_	Co_**1**_Dico_**2**_	Co_**2**_Dico_**2**_
Young’s modulus Co	110GPa	220GPa	110GPa	220GPa
Young’s modulus Dico	25GPa	25GPa	50GPa	50GPa

**Table 5:** Parameter combinations for cohesive parameter study.

Label	$G_{1}T_{0.1}$	$G_{1}T_{1}$	$G_{1}T_{4}$	$G_{4}T_{0.1}$	$G_{4}T_{1}$	$G_{4}T_{4}$
$G_{c}$ in $10^{3}\;J/m^{2}$	1.0	1.0	1.0	4.0	4.0	4.0
$T_{0}$ in MPa	0.1	1.0	4.0	0.1	1.0	4.0

**Table 6:** Water uptake for each conditioning state in mass percent.

Label	Dried	RC	Moist	Wet
Conditioning	50 °C 0% r.H.	23 °C 45% r.H.	50 °C 80% r.H.	50 °C submersed
Water uptake	0.00%	0.99%	2.00%	6.35%

**Table 7:** Run time performance of different mesh densities.

Mesh Density	Coarse	Medium	Fine
Run time	9981s	18,238 s	78,203 s
Multiple of coarse run time	-	1.83	7.84

**Table 8:** Run time performance of mass scaling factors.

Mass Scaling Factor	256	64	16	4	1
Run time	10,554 s	16,243 s	42,217 s	83,807 s	168,506 s
Run time factor to MS256	1.00	1.54	4.00	7.94	15.97

## Data Availability

All data are presented in the article.

## List of Abbreviations

CDP	Climbing Drum Peel
CERR	Critical Energy Release Rate
CF	Carbon Fiber
Co	Continuous
CoDico	Conitnuous-Discontinuous
DCB	Double Cantilever Beam
Dico	Discontinuous
FRP	Fiber Reinforced Polymer
FRTP	Fiber Reinforced Thermoplastic
LFT	Long Fiber Thermoplastic
LFT-D	LFT Direct
MS	Mass Scaling
PA 6	Polyamide 6
PTFE	Polytetrafluoroethylene
RC	Room Climate
UD	Unidirectional
